# Edge-Energy-Driven Growth of Monolayer MnI_2_ Islands on Ag(111): High-Resolution Imaging and Theoretical Analysis

**DOI:** 10.1021/acsnano.4c12146

**Published:** 2025-01-06

**Authors:** Daniel Rothhardt, Christopher Penschke, Hans Josef Hug, Regina Hoffmann-Vogel, Amina Kimouche

**Affiliations:** † Magnetic & Functional Thin Films Laboratory, Empa, Swiss Federal Laboratories for Materials Science and Technology, Ueberlandstrasse 129, 8600 Dübendorf, Switzerland; ‡ Department of Physics, University of Basel, CH-4056 Basel, Switzerland; § Institute of Physics and Astronomy, University of Potsdam, 14476 Potsdam-Golm, Germany; ∥ Institute of Chemistry, University of Potsdam, 14476 Potsdam-Golm, Germany

**Keywords:** edge energy, Hartree potential, local work function, scanning probe microscopy, Kelvin probe force microscopy, metal dihalides, magnetic two-dimensional materials, 2D materials

## Abstract

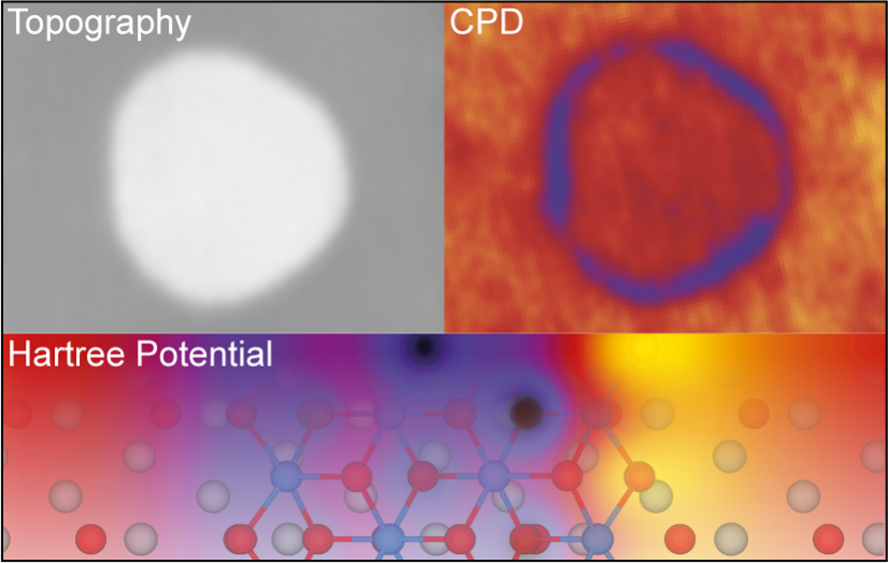

The reduced dimensionality of thin transition metal dihalide films on single-crystal surfaces unlocks a diverse range of magnetic and electronic properties. However, achieving stoichiometric monolayer islands requires precise control over the growth conditions. In this study, we employ scanning probe microscopy to investigate the growth of MnI_2_ on Ag(111) via single-crucible evaporation. The catalytic properties of the Ag(111) surface facilitate MnI_2_ dehalogenation, leading to the formation of a reconstructed iodine adlayer that acts as a buffer layer for the growth of truncated hexagonal MnI_2_ islands. These islands exhibit alternating edge lengths and distinct Kelvin potentials, as revealed by Kelvin probe force microscopy. Density functional theory (DFT) calculations support the experimentally observed island heights and lattice parameters and provide insights into the formation energies of both pristine and reconstructed edges. The asymmetry in edge lengths is attributed to differences in edge formation energies, driven by the position (up or down) of edge iodine atoms, as confirmed by DFT. This structural difference accounts for the observed variation in the Kelvin potential between the two types of island edge terminations.

## Introduction

The reduction in dimensionality in two-dimensional (2D) materials gives rise to distinctive properties that significantly differ from those of the conventional three-dimensional (3D) counterpart, making them potentially useful for nanoscale devices.^1−7^ Similar to the distinct characteristics of 3D material surfaces, the edges of 2D materials exhibit unique electronic, magnetic, optical, and catalytic properties compared to their 2D bulk.^8−17^ Pristine edges often undergo various reconstructions to enhance stability by rearranging atomic positions, which reduces dangling bonds and lowers the formation energy.^18^ So far, pristine and reconstructed edges have been observed in materials such as graphene,^19^ hexagonal boron nitride,^20^ black phosphorus,^21^ and transition metal dichalcogenides (TMDCs);^22^ however, they have not been reported in transition metal dihalides (TMDHs). TMDHs have garnered significant interest due to the recent discovery of magnetically ordered phases^23−29^ and their comparatively straightforward growth on metallic and superconducting substrates.^29−37^ Most 2D materials, including TMDCs, require intricate deposition techniques—such as MBE coevaporation,^38^ controlled atmospheric conditions during metal evaporation,^31^ physical vapor deposition^39,40^ or chemical vapor deposition^41,42^—to produce stoichiometric and atomically flat thin films. These processes typically involve extensive calibration of the precursor ratio and precise adjustments of the deposition parameters. A significantly simpler alternative is to employ single-source evaporation at relatively low evaporation temperatures of a high-purity stoichiometric TMDH powder.^32−37^ This approach, as first demonstrated by Zhou,^32^ bypasses the need for complex calibrations and the use of high-temperature evaporation of high melting point metals, permitting an efficient and reproducible fabrication of high-quality 2D TMDH layers under ultrahigh vacuum (UHV) conditions. Among these TMDHs, the wide gap semiconductor manganese(II) iodide (MnI_2_) is a particularly interesting material because it undergoes successive magnetic phase transitions upon cooling from room temperature and also develops a ferroelectric phase, making it a multiferroic material.^43−45^ The growth of MnI_2_ was first reported by Cai et al.,^31^ where manganese was evaporated in an iodine atmosphere to synthesize MnI_2_ on highly oriented pyrolytic graphite (HOPG) and subsequently the resulting surface was imaged using scanning tunneling microscopy (STM).

Here, we report the epitaxial growth of MnI_2_ monolayers on an Ag (111) substrate, achieved through thermal evaporation of stoichiometric MnI_2_ from a single glass crucible. We use STM and scanning force microscopy (SFM) to monitor the growth and analyze atomic-scale structures and phases of the MnI_2_ island formation process. The surface morphology undergoes substantial transformation as the substrate temperature is increased during evaporation. Initially, triangular islands evolve into hexagonal structures. Kelvin probe force microscopy (KPFM) measurements reveal pronounced changes in the local contact potential difference (LCPD) between the long and short edges of the hexagonally shaped MnI_2_ islands. By comparing density functional theory (DFT) calculations of the Hartree potential for various possible edge terminations with high-resolution KPFM measurements, we identified specific island edge terminations and configurations.

## Results and Discussion

**Figure 1:**
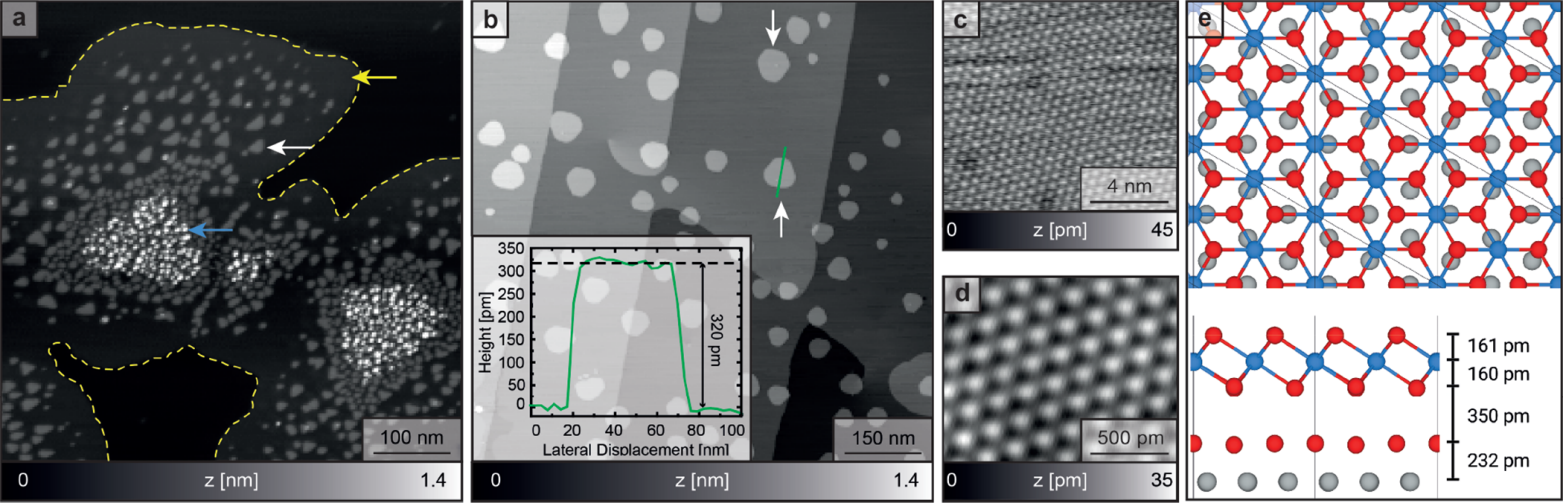
(a) SFM data of MnI_2_ on an iodine monolayer (yellow arrow) on a Ag(111) substrate. Smaller clusters (blue arrows) and MnI_2_ islands (white arrows) are visible. (b) STM data of MnI_2_ deposited onto Ag(111) at a substrate temperature of 395 K revealing hexagonal islands pointing downward and upward (white arrows). The inset shows a cross section through a MnI_2_ monolayer island with an apparent height of 330 pm at the location of the green line. (c) Zoomed SFM data obtained inside the larger islands are highlighted by the yellow arrow in (a). The atomic lattice constant corresponds to that expected for an iodine monolayer on the Ag(111) substrate. (d) STM data acquired on the hexagonal islands in (b) showing atomic resolution with a lattice constant compatible with MnI_2_. (e) DFT calculation showing the top and side views of a MnI_2_ monolayer on an iodine monolayer on a Ag(111) substrate. The filled red and blue circles represent iodine and manganese atoms, respectively. The faint lines indicate the boundaries of the supercell used in the calculation (corresponding to a 3 × 3 cell of Ag). The data in (a) and (c) were obtained with a frequency shift kept constant at −45 and −40 Hz, respectively, and an oscillation amplitude of 2 nm *f*_0_ = 191.51 kHz, *c*_L_ = 150 N/m. For (b) and (d), imaging parameters are 88 mV, 66 pA and 300 mV, 60 pA, respectively.

MnI_2_ islands were formed on a Ag(111) substrate using thermal evaporation. When the substrate was kept at room temperature, the formation of larger islands (Figure 1a yellow arrow and dashed outline) apart from triangular islands (Figure 1a white arrow) and a larger number of smaller protruding structures (Figure 1a pale-blue arrow) were observed. The small triangular islands are always found to be on top of the islands pointed out by the yellow arrow and the dashed outline. It is further interesting to note that the triangular islands point upward and downward in the left and right parts of the figure, respectively. Improved growth quality was achieved when the substrate temperature was kept at 395 K during the evaporation. The resulting surface depicted in Figure 1b reveals well-distributed islands of relatively homogeneous size with a hexagonal shape with alternating longer and shorter edges. Again, islands pointing upward and downward are observed (see white arrows in Figure 1b). Data with atomic-scale spatial resolution are obtained on the larger islands of Figure 1a (yellow arrow) as well as on hexagonal islands visible in Figure 1b (white arrow). The corresponding images reveal a hexagonal lattice with lattice parameters of 4.6 and 4.14 Å, displayed in Figure 1c,d, respectively.

The atomic-scale structure with the larger lattice constant (Figure 1c) agrees well with that observed in previous works^46,47^ studying a monolayer of atomic iodine thermally evaporated onto Ag(111). We thus conclude that the larger structure observed here, and the islands in Figure 1a outlined by the dashed yellow line, also consists of a  reconstruction of atomic iodine acting as a buffer layer for the successive MnI_2_ islands. In contrast to the work of Cai et al.,^48^ who evaporated Mn in an iodine atmosphere, where the latter was obtained by decomposing CrI_3_ in a crucible, we employed a single crucible to evaporate MnI_2_ at a moderate crucible temperature of 653 K. Previous works^32−37^ showed that the TMDHs do not decompose in the crucible at moderate temperatures as shown by X-ray photoemission spectroscopy and SPM. The formation of halide layers observed here and for NiBr_2_^33^ arises from the catalytic activity of noble metal surfaces leading to a dehalogenation of the TMDHs. Hence, the triangular and hexagonal islands visible in Figure 1a,b, respectively, grow exclusively on the atomic iodine layer. The two distinct orientations of the islands (white arrows in Figure 1b) are attributed to the two different domains of the iodine  reconstruction. The lattice parameter of 4.14 Å of the smaller structure in Figure 1d agrees well with both the experimental work discussing the growth of MnI_2_ islands on graphene^48^ and the DFT studies of a freestanding monolayer of MnI_2_ in a vacuum.^26,49^ Here, we have performed gradient-corrected DFT calculations involving a Hubbard correction of a MnI_2_ monolayer on an iodine layer on Ag(111) (Figure 1e). The lattice parameter is again 4.11 Å. We thus conclude that the observed triangular and hexagonal islands must consist of MnI_2_ growing on the Ag(111)-I reconstruction of the atomic iodine phase. Our DFT calculation displayed in Figure 1e (bottom panel) reveals a monolayer height of 671 pm of MnI_2_ grown on I/Ag(111), while the height obtained from STM is 320 pm (see the cross section displayed in the inset of Figure 1b). This is because the tip–sample distance in STM depends on the local density of states at the Fermi energy and the measured corrugation would only correspond to the real topography if the measurement was conducted on a homogeneous surface, which is clearly not the case here.

An improved island height assessment can be derived by advanced SFM techniques. To this end, a phase-locked loop is employed to oscillate an SFM cantilever with a PtIr-coated Si tip on its resonance frequency (*f*_0_ = 245.72 kHz and force constant *c*_*L*_ = 40 N/m) at an oscillation amplitude kept constant at 6 nm. During the measurement of the sample topography, a feedback adjusts the tip–sample distance to maintain the negative frequency shift Δ*f* arising from the overall attractive tip–sample interaction force gradient constant. The movement of the tip along the *z*-direction then represents contours of constant total , with the *z*-derivative of the *z*-component of the force weighted over the tip–sample distance range [*z*, *z* + 2*A*] being covered by the oscillation of the tip with an amplitude *A*.^50^

**Figure 2:**
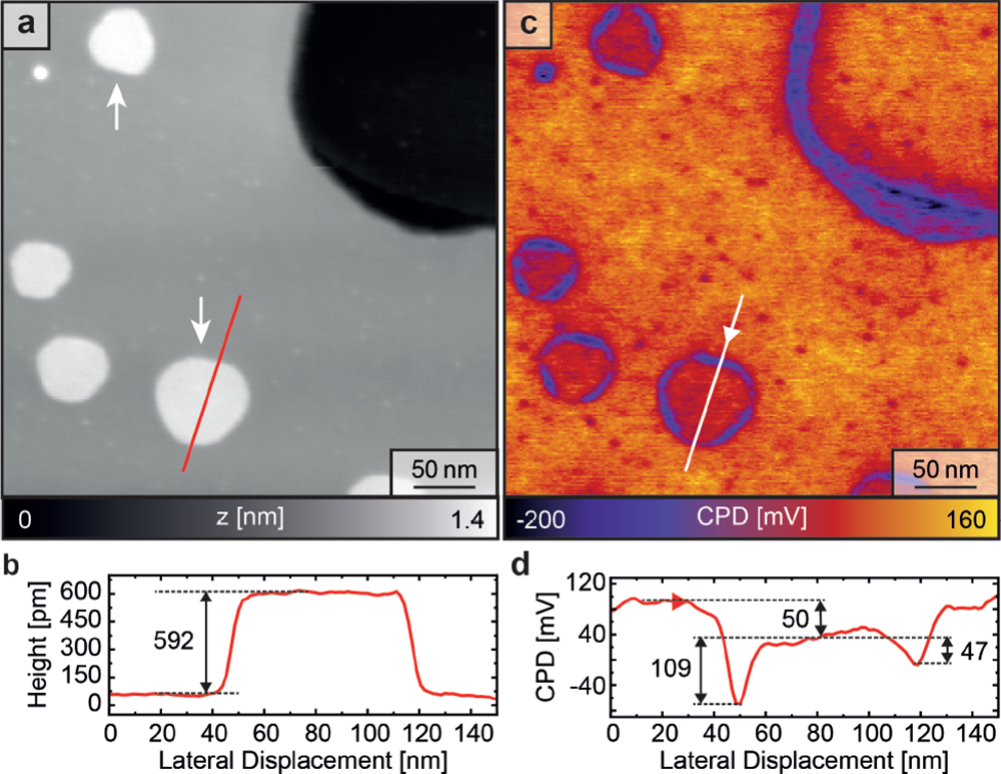
(a) SFM topography data obtained with locally compensated CPD at a frequency shift set point of −106 Hz showing hexagonal MnI_2_ islands on the iodine monolayer adsorbed on the Ag(111) substrate. (b) Cross section was taken at the red line in (a) across a MnI_2_ island showing a height of 592 pm. (c) LCPD data acquired simultaneously with (a) revealing LCPD differences between the MnI_2_ island centers and edges and the iodine monolayer. (d) LCPD cross section taken at the location (direction) of the green line (arrow) in (c) revealing LCPD differences between the iodine layer and the island center and between the island center and the upper and lower island edges.

The measured  arises from many different interactions. One of those, the long-range electrostatic force, depends quadratically on the difference between the applied bias voltage and the LCPD and can lead to a wrong height determination in SFM measurements. An approach to overcome this problem is to employ KPFM.^51−53^ KPFM is a method for minimizing local electrostatic forces arising from the LCPD. In frequency-modulated KPFM (FM-KPFM), a lock-in amplifier is used to modulate the cantilever frequency with an AC frequency ω_ac_ = 650 Hz, resulting in the appearance of side bands at positions *f*_0_ ± ω_ac_. A Kelvin feedback then adjusts the applied tip–sample potential to nullify the amplitudes at the (*f*_0_ ± ω_ac_)-side bands and hence compensates for the local CPD. The resulting image represents contours of the local CPD between the tip and the surface. The SFM topography, measured with a Δ*f*-set point of −106 Hz and a locally compensated CPD, is depicted in Figure 2a, showing large hexagonal MnI_2_ islands with alternating longer and shorter edges. Again, downward- and upward-pointing islands are observed (white arrows). The line profile in Figure 2b (red line in Figure 2a) shows a uniform island height of 592 ± 6 pm, which agrees with our DFT calculation results shown in Figure 1e (bottom panel). The small deviation in height between the experimental and DFT data can be attributed to differences in the van der Waals force gradients between the MnI_2_ island and the iodine adlayer on the Ag(111) surface. The MnI_2_ islands appear with a KPFM contrast that is about 50 mV smaller than that of the surrounding iodine layer (Figure 2c). Moreover, the LCPD values of the MnI_2_ island's short and long edges are 47 and 109 mV lower than that of the center of the island (Figure 2d).

The hexagonal shape of the MnI_2_ islands arises from the 6-fold symmetry of MnI_2_. The unequal length of alternating MnI_2_ island edges as well as their substantially different Kelvin potential must arise from a difference in energy of alternating edges.^54,55^ To explore possible terminations of island edges and their formation energies, DFT calculations of MnI_2_ ribbons suspended in vacuum and on the Ag(111)-I surface were performed.

**Figure 3:**
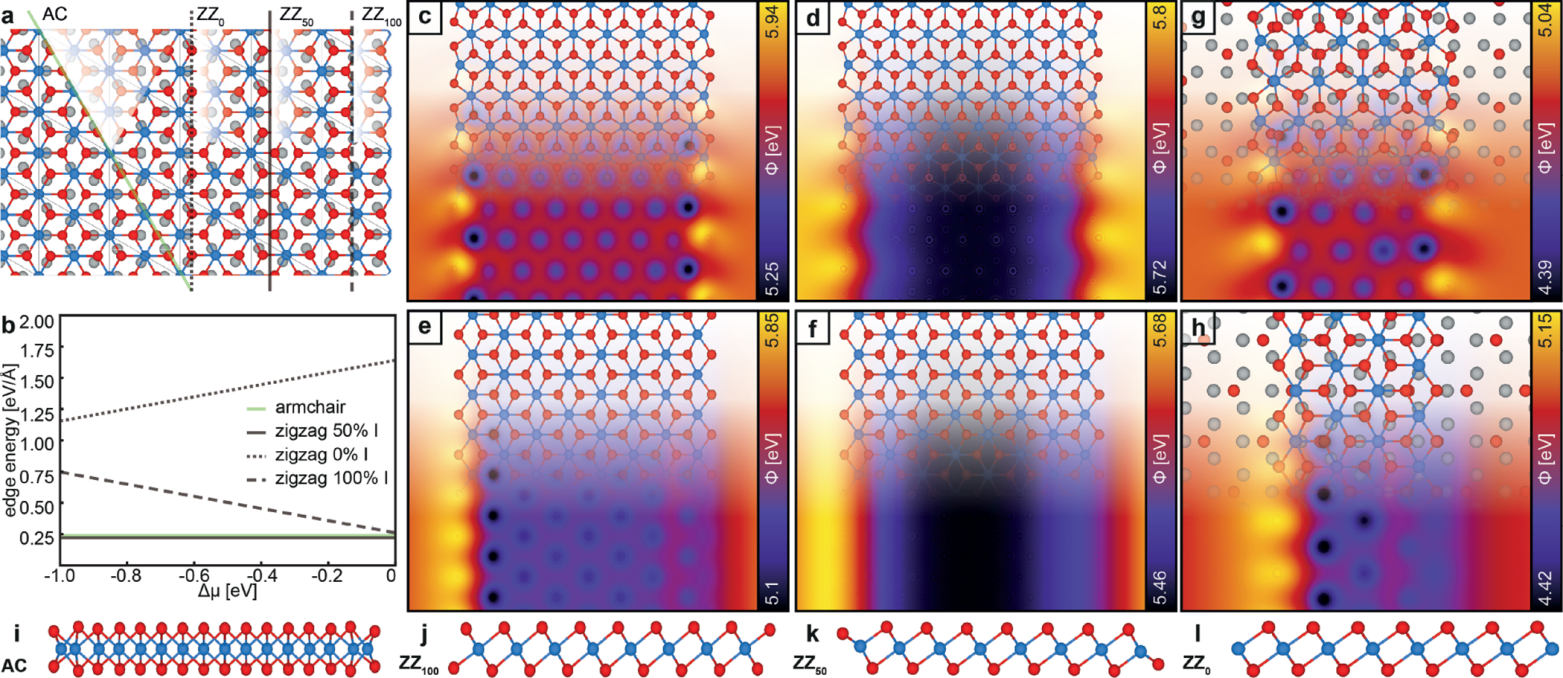
(a) Schematics of the MnI_2_ structure with AC (solid green), ZZ_0_ (dotted green), ZZ_50_ (dotted gray), and ZZ_100_ (solid gray line) edge terminations. The shaded regions next to the cutting lines highlight the visibility of the corresponding edge atoms. (b) Dependence of the edge energy per unit length for the different edge terminations on the chemical potential μ. Calculated local work functions ϕ for a MnI_2_ ribbon suspended in vacuum with the AC edge termination at distances *z* = 2 Å (panel c), *z* = 5 Å (panel d) and ZZ_50_ edge termination at distances *z* = 2 Å (panel e), *z* = 5 Å (panel f). Calculated local work functions ϕ for a MnI_2_ ribbon on the Ag(111)-I surface at a distance *z* = 2 Å for the AC (panel g) and ZZ_50_ (panel h) edge terminations. Side views of the different possible terminations of the left and right edges of MnI_2_ ribbons for (i) AC, (j) ZZ_100_, (k) ZZ_50_, and (l) ZZ_0_ edge terminations.

Like for PtSe_2_ on HOPG studied by Li et al.,^56^ cutting MnI_2_ islands along the armchair direction (light-green line in Figure 3a,i) results in only one possible edge termination (AC), while cutting along the zigzag direction leads to three different types of edge terminations, 0, 50, and 100% I (ZZ_0,50,100_ indicated by the dotted, solid, and dashed black lines in Figure 3a,j,k,l, respectively). However, only the AC and ZZ_50_ edges are stoichiometric terminations with a Mn:I ratio of 1:2, while the other edge terminations such as the ZZ_100_ and ZZ_0_ edge terminations are nonstoichiometric, containing too much or too little iodine, respectively, and are thus expected to be less stable. In DFT model calculations, such terminations can be forced to exist by removing and adding iodine to the ZZ_50_ edge, represented by the chemical potential μ_I_ defined relative to the “0 K; 0 bar” reference state of the DFT calculations. The energies of the stoichiometric AC and ZZ_50_ edge terminations are 0.24 and 0.22 eV/Å (green and solid gray lines in Figure 3b, respectively), independent of the chemical potential. The edge energies of ZZ_100_ and ZZ_0_ linearly decrease and increase with the chemical potential. ZZ_100_ is close in energy at a high chemical potential, but at values of μ_I_ expected under the typical experimental conditions, it is much higher in energy. The energy of ZZ_0_ is much higher than those of the other terminations over a wide range of chemical potential values. We thus conclude that the hexagonal MnI_2_ islands are terminated by either AC or ZZ_50_ edges because these have the lowest energies. To understand the experimentally observed difference in the CPD of adjacent edges, calculations on the local work function of a MnI_2_ ribbon with different edge terminations were performed. While the Kelvin potential is defined as the energy to remove an electron from the Fermi energy level, the energy to remove an electron from an atomic-scale object generally depends on the spatial location^57^ as described by the Hartree potential *H*(*x*, *y*, *z*). The *z*-dependence arises from the exponential decay of the electrostatic fields with the k-vector given by the in-plane wavelength and thus the *xy*-dependence of the Hartree potential. To obtain the local work function for a MnI_2_ ribbon, the difference in the Hartree potential and Fermi energy is calculated. The results for the AC terminated MnI_2_ ribbon suspended in vacuum are depicted in Figure 3c,d for heights *z* = 2 and *z* = 5 Å, respectively. Compatible with the experimental results, a lower work function is observed at the ribbon (island) center compared with the ribbon edges. The data for *z* = 2 Å (Figure 3c) shows a pronounced atomic-scale variation (with the lower work function at the iodine atoms visible as purple spots) and the same contrast on the left and right edges, while for *z* = 5 Å, a small contrast difference of only about 9 meV is observed. Note that such an absent or very small difference between opposite edges significantly deviates from the experimentally observed difference of 109 meV (Figure 2d). While the results for a MnI_2_ ribbon with ZZ_50_ edge termination again show a lower work function at the ribbon center compared to the ribbon edge, the differences between the left and right edges are about 160 and 60 meV for *z* = 2 and *z* = 5 Å, respectively, agreeing well with the experimentally observed value. Note, however, that the DFT results depicted in Figure 3c–f were obtained for MnI_2_ ribbons in a vacuum. Improved modeling with the MnI_2_ ribbon on the Ag(111)-I surface was performed for the ZZ_50_ edge termination. The results depicted in panels g and h reveal that the overall level of the work function is slightly reduced by the iodine adlayer, but differences in contact potentials between the left and right edges persist. We thus conclude that the MnI_2_ islands are terminated with ZZ_50_ edges. Further, we attribute the observed substantial difference in work function to the iodine atoms on the left and right edges of a ZZ_50_ terminated MnI_2_ ribbon pointing away (up) and toward (down) the surface, respectively.

**Figure 4:**
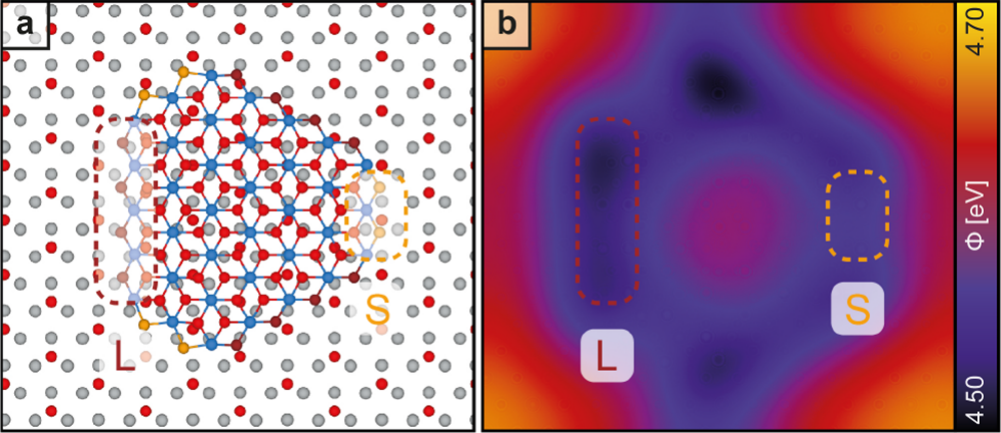
(a) Structure of a hexagonal MnI_2_ “island” with ZZ_50_ edges on iodine on Ag(111) optimized by DFT. Orange and purple dots represent iodine edge atoms pointing upward and downward, respectively. The dotted red and yellow boxes highlight the opposite long (L) and short (S) edges. (b) Calculated local work function at a distance *z* = 5 Å, with the dotted red and yellow boxes highlighting the difference of the work functions between the long and short edges.

In addition to the ribbon model illustrated in Figure 3, we calculated the local work function of hexagonal islands on the I reconstruction. The structure used for this calculation is shown in Figure 4a, where the edge iodine atoms are highlighted with different colors. The resulting local work function map, presented in Figure 4b, reveals that the island center exhibits a higher work function compared to the edges, while it is slightly lower than that of the surrounding iodine adlayer. Notably, the longer edges, where the iodine atoms are pointing downward, demonstrate the lowest work function, which correlates well with the KPFM data shown in Figure 2c. The edge structure is also expected to influence the edge energies and, consequently, the relative lengths of opposite edges. Our DFT calculation of the edge energies of MnI_2_ islands on an iodine monolayer on Ag(111) reveals an energy difference (Δ*E*) of 29 meV per atom between the upward- and downward-pointing (lower energy) iodine edge atoms. To estimate the ratio of the lengths of opposite edges, the ratio of the occupation numbers of these two distinct states can be obtained using a Boltzmann distribution:
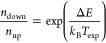
1where *n*_down_ and *n*_up_ are the numbers of downward- and upward-pointing iodine edge atoms, respectively. For *T*_exp_ = 395 K, which is the Ag(111) substrate temperature during MnI_2_ evaporation, a ratio of 2.34 is obtained. However, an analysis of multiple islands shows the ratio between the different edge lengths to be 1.99 ± 0.05. Note that the calculation was performed for zero temperature, while the MnI_2_ islands formed at a substrate temperature of 395 K used during the evaporation. We expect that at this temperature, the inherent positional fluctuations of the edge iodine atoms around the equilibrium positions lower the energy difference between terminations of opposite edges (i.e., upward- and downward-pointing iodine atoms) and will thus lead to a reduced ratio between the lengths of nonequivalent ZZ_50_ edges compared to that obtained from DFT considering a zero-temperature situation.

## Conclusions

In conclusion, we have demonstrated the growth of 2D MnI_2_ on Ag(111) by thermally evaporating a stoichiometric MnI_2_ powder from a single crucible. However, the catalytic nature of the Ag(111) surface leads to partial dehalogenation of MnI_2_, resulting in a reconstructed iodine layer, atop which MnI_2_ islands form. These islands exhibit a hexagonal shape with edges of unequal lengths and LCPD. The unequal edge lengths stem from the edge formation energy differences between distinct edge terminations, which also explain the observed difference in Kelvin potentials.

Given that TMDHs generally exhibit greater thermal stability than their trihalide counterparts, our work suggests that single-crucible evaporation offers a convenient approach for growing 2D TMDH systems on single-crystalline substrates. Among the latter are noble metal surfaces, which typically show a pronounced catalytic activity leading to dehalogenation. For future work, we propose that this dehalogenation effect could be suppressed by first coating the substrate with the corresponding halogen layer, which may also serve as a layer to decouple the 2D TMDHs from the substrate.

## Experimental Section and Methods

A one-side-polished Ag(111) single crystal (MaTeck) was prepared by repeated cleaning cycles in UHV. Each cycle consists of Ar^+^-ion bombardment (*P*_Ar_ = 2 × 10^–5^ mbar, *I*_ion_ = 5 μA, *V*_ion_ = 0.8 kV, *t* = 15 min) followed by annealing up to 750 K for 15 min. The surface quality was checked using a scanning probe microscope (SPM). A three-pocket evaporator (Kentax GmbH) was used to evaporate MnI_2_ (Sigma-Aldrich) at an evaporation temperature of *T* = 653 K. Some samples were annealed to 395 K prior to and during the evaporation process to optimize the growth quality. SPM measurements were carried out using a variable-temperature scanning probe microscope (VT-SPM, Scienta Omicron) operated at 120 K under UHV conditions with a base pressure of 2 × 10^–10^ mbar.

All DFT calculations were performed using VASP,^58,59^ version 5.4.4. The PBE functional^60^ was employed together with the D3 dispersion correction^61^ with Becke–Johnson damping.^62^ An effective Hubbard U parameter of 4 eV was applied to the Mn d states, which has been suggested by Wu et al.,^63^ based on comparison to experimental results. Lattice parameters were optimized using bulk models, resulting in values of 411 and 288 pm for MnI_2_ and Ag, respectively. The Ag(111) surface was modeled using a three-layer slab, with atoms in the lowest layer fixed at bulk positions. The self-consistent field method and force convergence criteria were set to 10^–6^ eV and 10^–2^ eV/Å, respectively. The calculation of a complete MnI_2_ layer on I/Ag(111) shown in Figure 1e was performed on a 3 × 3 supercell of Ag (863 × 863 pm) with a 6 × 6 × 1 k-point grid. Calculations on the MnI_2_ edge terminations used 10 k-points along the periodic direction. For ZZ_50_ and AC on Ag(111), 6 × 6 (1.726 nm × 2.990 nm) and 12 × 3 (3.452 nm × 1.495 nm) supercells were used, respectively, which were sampled with k-point grids of 2 × 1 × 1 and 1 × 2 × 1, respectively. The structure shown in Figure 4 used a 15 × 8 supercell (4.315 nm × 3.986 nm), which was calculated at the Γ point only. The vacuum height was at least 1.1 nm for structure optimizations and was increased to at least 1.5 nm for the calculations of the local potential.

The local work function Φ (*x*, *y*, *z*) was determined by subtracting the Fermi energy from the Hartree potential, i.e., Φ (*x*, *y*, *z*) = *V*_Hartree_(*x*, *y*, *z*) – *E*_Fermi_. Two-dimensional maps were obtained for planes parallel to the surface at heights *h* of 0.2 and 0.5 nm relative to the uppermost atom of the MnI_2_ layer (i.e., Φ_*h*_ (*x*, *y*) = Φ (*x*, *y*, *z*_max_ + *h*). The heights for the local work function were chosen to either show the work function at the surface in detail (0.2 nm) or represent typical distances in the experiment (0.5 nm). A height of 1.0 nm was also tested, which showed results comparable to 0.5 nm.

Edge energies are calculated as 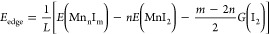
with *L* as the unit cell length along the edge, *E*(Mn_*n*_I_*m*_) as the energy of a ribbon with *n* Mn and *m* I atoms, *E*(MnI_2_) as the energy of an ideal three-dimensional MnI_2_ layer, and *G*(I_2_) = *E*(I_2_) + 2Δ μ_I_ as the Gibbs free energy of I_2_ (given as the DFT energy of an I_2_ molecule plus the change in the chemical potential of I).
